# Radiotherapy for Locally Advanced Pancreatic Adenocarcinoma—A Critical Review of Randomised Trials

**DOI:** 10.3390/curroncol30070499

**Published:** 2023-07-18

**Authors:** Mathilde Weisz Ejlsmark, Tine Schytte, Uffe Bernchou, Rana Bahij, Britta Weber, Michael Bau Mortensen, Per Pfeiffer

**Affiliations:** 1 Department of Oncology, Odense University Hospital, 5000 Odense, Denmark; 2 Department of Clinical Research, University of Southern Denmark, 5000 Odense, Denmark; 3 Laboratory of Radiation Physics, Odense University Hospital, 5000 Odense, Denmark; 4 Department of Oncology, Aarhus University Hospital, 8200 Aarhus, Denmark; 5 Danish Centre of Particle Therapy, Aarhus University Hospital, 8200 Aarhus, Denmark; 6 Department of Surgery, Odense University Hospital, 5000 Odense, Denmark

**Keywords:** pancreatic adenocarcinoma, radiotherapy, chemoradiation, stereotactic body radiation therapy, ablative radiotherapy, MR-guided radiation therapy

## Abstract

Pancreatic cancer is rising as one of the leading causes of cancer-related death worldwide. Patients often present with advanced disease, limiting curative treatment options and therefore making management of the disease difficult. Systemic chemotherapy has been an established part of the standard treatment in patients with both locally advanced and metastatic pancreatic cancer. In contrast, the use of radiotherapy has no clear defined role in the treatment of these patients. With the evolving imaging and radiation techniques, radiation could become a plausible intervention. In this review, we give an overview over the available data regarding radiotherapy, chemoradiation, and stereotactic body radiation therapy. We performed a systematic search of Embase and the PubMed database, focusing on studies involving locally advanced pancreatic cancer (or non-resectable pancreatic cancer) and radiotherapy without any limitation for the time of publication. We included randomised controlled trials involving patients with locally advanced pancreatic cancer, including radiotherapy, chemoradiation, or stereotactic body radiation therapy. The included articles represented mainly small patient groups and had a high heterogeneity regarding radiation delivery and modality. This review presents conflicting results concerning the addition of radiation and modality in the treatment regimen. Further research is needed to improve outcomes and define the role of radiation therapy in pancreatic cancer.

## 1. Introduction

The burden of cancer, including pancreatic cancer (PC), is growing worldwide, not only due to ageing and the growth of the population, but also due to changes in risk factors like diabetes [1]. The prognosis for patients with PC is dismal, with a 5-year survival rate of only 3–8%, and PC accounts for almost as many deaths (466,000) as cases (496,000 worldwide per year) [1,2,3]. PC is projected to become the second leading cause of cancer deaths in the US and third in Europe by 2025 [1,4].

Around half of the patients with PC, which in this review means pancreatic ductal adenocarcinoma (PDAC), are diagnosed with metastatic disease (mPC). Patients without metastasis may be staged according to the TNM classification, but more often, they are divided into three major clinical relevant groups of approximately similar size: resectable disease (rPC), borderline resectable (brPC), and locally advanced (LAPC) disease. This division is based on the degree of contact between the tumour and adjacent vessels/organs [5,6,7]. A multidisciplinary approach (including experts in surgery, medical oncology, radiotherapy, radiology, and pathology) is the optimal strategy for staging, classification, and selecting the best treatment strategy for patients with PC.

Resection offers a chance for a cure for patients with rPC. Resection for brPC should be preceded by preoperative chemotherapy to control potential micro-metastatic disease but occasionally also to shrink the tumour [5,7]. In this review, we define neoadjuvant therapy as preoperative treatment for patients with upfront resectable PC. For patients with LAPC, the purpose of conversion (or induction or down-sizing) therapy is to shrink the tumour to facilitate resection in patients with initial non-resectable disease.

Several randomised trials have tested neoadjuvant therapy before surgery (with or without supplementary adjuvant therapy) to upfront surgery (with or without supplementary adjuvant therapy) in patients with rPC and/or brPC [8,9,10,11,12,13,14]. Four trials tested chemoradiation, two trials tested chemotherapy alone, and a small four-arm study compared chemo- and radio-therapy (the ESPAC-5 study) [14]. None of the trials used the modern regimen called modified FOLFIRINOX (folinic acid, 5FU, irinotecan, oxaliplatin) as postoperative therapy, which currently is considered the standard of care. Three of the trials testing chemoradiation did not reach their accrual targets. However, the median overall survival was numerically longer in all trials testing preoperative therapy. Several meta-analyses have subsequently been conducted [15,16,17]. A significant prolonged overall survival was mainly found in the subgroup of patients with brPC (i.e., venous contact >180 degrees or any arterial contact). Subgroup analyses found improved overall survival rates for both preoperative chemoradiation (HR 0.74) and chemotherapy (HR 0.54). Updated results from the PREOPANC trial showed that the long-term overall survival improved for patients with rPC and brPC treated with neoadjuvant gemcitabine-based chemoradiation [18].

Adjuvant radiotherapy or chemoradiation is not recommended and should not be offered following surgery outside the setting of clinical trials [19].

Based on data from randomised studies, the expected median overall survival after resection and adjuvant gemcitabine is around 20 months, with a five-year overall survival of approximately 20%, though the smaller the tumour, the greater the chance of long-term survival. Adjuvant gemcitabine-based chemotherapy increased the chance for long-term survival, and modified FOLFIRINOX has improved results even further with an overall survival rate at five years of 43.2% compared to 31.4% with gemcitabine monotherapy [20]. With increasing size and vessel involvement (brPC and LAPC), the median overall survival has become progressively shorter. With gemcitabine as the standard of care, the median overall survival is approximately six months for patients with non-resectable PC. Still, the introduction of modern combination chemotherapy has significantly prolonged the median overall survival to 9–11 months, with tolerable toxicity [20,21]. In contrast, the median overall survival for patients without oncological therapy is only a few months [22].

Remarkable improvements have been made in the last ten years, particularly regarding systemic therapy and radiation delivery techniques [5,7]. However, when evaluating and comparing results from radiation trials, one must also consider that the overall strategy has changed from radiotherapy to chemoradiation and to induction combination chemotherapy before chemoradiation. Furthermore, radiotherapy has improved from the box technique to 3D conformal radiation and intensity-modulated radiation therapy (IMRT), with or without concomitant chemotherapy, and most recently, with stereotactic body radiation therapy (SBRT) using small fields and high doses guided by daily imaging. In addition, radiotherapy plan adaption based on the anatomy of the day is now possible with the introduction of online magnetic resonance (MR) image-guided treatment techniques [23]. A decade ago, the median overall survival for LAPC patients was around 12 months. However, in recent studies with selected patients, the median overall survival has increased to or even surpassed 18 months [7,24].

Patients with LAPC have an intermediate prognosis between those with resectable and metastatic disease, with a median overall survival of around 12–15 months determined in recent overviews [25]. The chemotherapy results alone are difficult to evaluate in the literature since many trials conducted before the landmark FOLFIRINOX trial pooled patients with LAPC and metastatic cancer [26,27]. Recent research has also shown that combination chemotherapy may shrink the primary tumour to allow subsequent resection, which may then be curative in selected cases [28]. Presently, it is not established whether the optimal therapy in LAPC is chemotherapy or chemoradiation, but the sequence of combination chemotherapy followed by chemoradiation is so far the most promising and most widely used [25,29]. The purpose of any therapy is to delay progression, prolong overall survival, and preserve or improve quality of life.

Since the publication of the Burris trial, chemotherapy has been an established integrity of standard therapy in many patients with PC [30]. In contrast, the advantage of radiotherapy is still heavily debated, even though radiotherapy was introduced long before the gemcitabine era.

In the present paper, we will review data on radiotherapy in patients with mainly non-resectable pancreatic ductal adenocarcinoma (PDAC), knowing that there is, to some extent, an overlap between brPC and LAPC. We will cover standard fractionated radiotherapy (sRT), chemoradiation, and SBRT.

## 2. Materials and Methods

### Search Strategy

**Figure 1:**
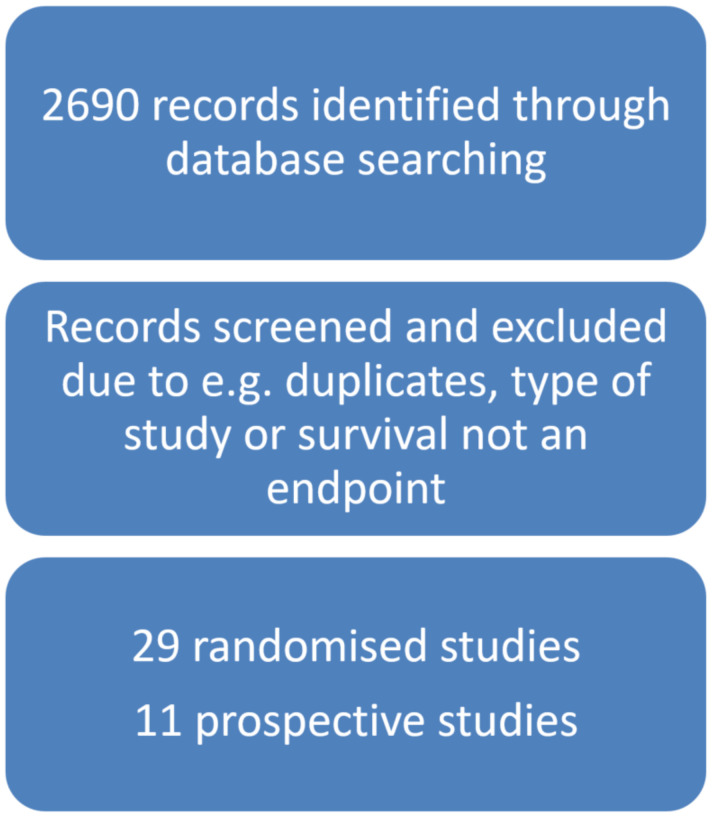
Flowchart of the search strategy.

A regular search of Embase and the PubMed database was conducted, focusing on studies involving LAPC (or non-resectable PC) and radiotherapy without any limitation for the time of publication. Our search queried keywords were “pancreatic adenocarcinoma” OR “pancreatic cancer” OR “pancreatic neoplasm” AND “radiotherapy” OR “chemoradiotherapy” OR “stereotactic body radiation therapy (SBRT) OR “stereotactic ablative radiotherapy (SABR)”, see Supplementary A. English language articles with information on doses, fractionation, and outcome were included in this review. The references of selected papers and previous systematic reviews and meta-analyses were checked for further references to other relevant trials. Articles were selected based on their abstract. We included randomised controlled trials involving patients with brPC or LAPC, including radiotherapy or chemoradiation or SBRT with or without chemotherapy before or after radiotherapy. At least one treatment arm should evaluate radiotherapy. Our findings are demonstrated in Figure 1.

In this review, we use the term locally advanced pancreatic ductal adenocarcinoma for patients with non-resectable pancreatic cancer, defined as a tumour that has invaded neighbouring structures, such as large vessels, without any sign of metastasis to distant organs. The term locally advanced pancreatic cancer has been modulated over time due to slight changes in the definition and criteria of staging PDAC [6]. Whenever possible, we have separated patients in brPC and LAPC. Furthermore, we allocated studies according to the following four major sub-groups: early studies with radiotherapy versus chemoradiation; which drug to use as sensitiser; chemotherapy or chemoradiation; and SBRT.

## 3. Results

### 3.1. Early Studies with Radiotherapy and Chemoradiation

Before the introduction of multi-detector computed tomography (CT) angiography, accurate preoperative staging was not possible. Non-resectability due to arterial and/or venous involvement was, therefore, often verified during a laparotomy [31]. Is it possible to extract relevant data from early randomised studies conducted before the use of modern radiation techniques and before chemotherapy became the standard of care?

Initial studies were mainly conducted by US/Canadian institutions/groups. Studies were often with a limited number of patients, some patients were excluded for various reasons, and outdated radiation techniques and schedules, like the box technique and split-course therapy, were tested. However, a few trials and aspects deserve special attention.

As early as 1969, a small randomised trial showed that radiation with chemotherapy (concurrent bolus 5FU—chemoradiation) was more effective than radiation alone [32]. The 1981 GITSG study confirmed the benefit of 5FU-based chemoradiation [33]. After including 106 patients, the radiation-alone arm was closed due to an inferior overall survival of just 5.3 months compared to around 10 months in the two chemoradiation arms. There was a trend (median overall survival 8.4 and 11.4 months, respectively), but no significant difference in the median overall survival comparing 40 Gy and 60 Gy split-course therapy (planned treatment break of around two weeks after two weeks radiotherapy).

The median overall survival was very short in all studies (less than 12 months), but patients receiving chemoradiation had a longer median overall survival. An average of 37 patients were included in each treatment arm in the 11 randomised trials presented in Table 1, and patients receiving radiotherapy or chemoradiation had an average overall survival of 8.5 months.

Standard, conventionally fractionated radiotherapy, delivering 40 to 60 Gy in 1.8–2.0 Gy per fraction, has had a modest, if any, impact on patients with LAPC. These doses were used due to limitations posed by the tolerability of organs at risk (OAR) in the abdomen at a time when two-dimensional planning necessitated larger treatment volumes to account for an increased uncertainty in target definition and treatment delivery [34].

However, in a small (n = 31) but very important Japanese trial, chemoradiation (radiotherapy 50.4 Gy/28 fractions with concomitant continuous infusion of 5FU), compared to the best supportive care (BSC), significantly prolonged the median overall survival from 6.4 months to 13.2 months (*p* = 0.001) in patients with LAPC. The one-year survival was increased from 0% to 53%, and quality of life (QoL) was improved [35]. However, it is still an open question whether the same advantage would have been achieved with chemotherapy alone (5FU or gemcitabine monotherapy).

Another small but also noteworthy Japanese study [36] included 42 patients with resectable tumours (no involvement of the celiac axis or the superior mesenteric artery). At laparotomy, but before resection, patients were randomised into a resection group and a chemoradiation group (50.4 Gy/28 fractions with 5FU 200 mg/m${}^{2}$/day infusion) without resection. There was no major difference in the median overall survival (9 vs. 12 months), but more patients in the resection group were alive after three years (0 vs. 30%) and five years (0 vs. 10%), respectively [37]. No patient receiving chemoradiation in the two Japanese trials was alive at 24 months, and it may be concluded that conventional chemoradiation (around 50 Gy in 2 Gy fractions) has no curative potential on its own.

**Table 1:** Radiotherapy or chemoradiation in patients with non-resectable pancreatic cancer—summary of randomised trials.

	Institution or Group	Phase	Therapy	Radiotherapy Gy/fx	n	Median OS Months	1Y OS %
Childs [38]	Mayo	III	RT	35–40 Gy/20 fx	12	5.4	11
Radiology 1965			CRT (5FU bolus)		13	7.0	31
Moertel [32]	Mayo	III	RT	35–40 Gy/20 fx	32	6.3	5
Lancet 1969			CRT (5FU bolus)	35–40 Gy/20 fx	32	10.4	25
Hazel [39]	Canada	III	5FU (+CCNU)		15	7.8	-
JCAR 1981			CRT (5FU) ⟹ CCNU	46 Gy/23 fx	15	7.8	-
Moertel [33]	GITSG	III	RT	60 Gy/30 fx split	25	5.3	12
Cancer 1981			CRT (5FU) ⟹ 5FU	40 Gy/20 fx split	83	8.4	35
			CRT (5FU) ⟹ 5FU	60 Gy/30 fx split	86	11.4	47
Klaassen [40]	ECOG	III	5FU		44	8.2	28
JCO 1985			CRT (5FU)	40 Gy/20 fx	47	8.3	30
GITSG [41]	GITSG	III	CRT (5FU) ⟹ 5FU	60 Gy/30 fx split	73	8.5	33 ^1^
Cancer 1985			CRT (Adr) ⟹ Adr	40 Gy/20 fx split	70	7.5	27 ^1^
GITSG [42]	GITSG	III	SMF		22	8.0	-
JNCI 1988			CRT (5FU)⟹ SMF	54 Gy/30 fx	21	10.5	-
Earle [43]	GITSG	III	CRT (5FU)	60 Gy/30 fx split	44	7.8	35
IJROBP 1994			CRT (Hyc)	50 Gy/25 fx split	43	7.8	28
Shinchi [35]	Japanese	III	BSC		15	6.4	0
IJROBP 2002			CRT (5FU)	50.4 Gy/28 fx	16	13.2	55
Imamura [36]	Japanese	III	CRT (5FU)	50.4 Gy/28 fx	22	9	32
Surgery 2004			Surgery		20	13	62
Cohen [44]	ECOG	III	RT	59.4 Gy/33 fx	49	7.1	20 ^1^
IJROBP 2005			CRT (5FU-MMC)	59.4 Gy/33 fx	55	8.4	32 ^1^

^1^ Estimated 1 year overall survival.

Two meta-analyses on LAPC concluded that chemoradiation prolonged the median overall survival compared with radiotherapy alone, but chemoradiation followed by chemotherapy did not lead to a survival advantage over chemotherapy alone [45,46]. Most trials in Table 1 were included in a Cochrane meta-analysis [45]. The identified randomised studies were too heterogeneous (radiation schedules and techniques and chemotherapy employed) for a pooled analysis. The authors performed a qualitative overview and concluded that chemoradiation appears to have a benefit over radiotherapy alone. In addition, they concluded that there is insufficient evidence to recommend chemoradiation as a superior alternative to chemotherapy alone in patients with LAPC. 

Conclusions

- Chemoradiation is more effective than radiotherapy alone for non-resectable PC.
- Chemoradiation prolongs the overall survival compared to best supportive care.

### 3.2. Which Drug to Use as a Sensitiser

Before the millennium, trials showed that 5FU-based chemoradiation was more effective than radiotherapy alone. However, 5FU-based chemoradiation was challenged by a small study in which 34 patients with LAPC were randomised to three-dimensional conformal radiotherapy (3D-RT) plus weekly gemcitabine or 5FU as a continuous infusion [47]. All patients received maintenance gemcitabine after chemoradiation until progression. Chemoradiation with gemcitabine increased the response rate (50% versus 12%), improved the median progression-free survival from 2.7 to 7.1 months, and prolonged the median overall survival from 6.7 months to 14.5 months (*p* = 0.02). One may argue that the progression-free survival and overall survival in patients receiving chemoradiation with 5FU are, for unknown reasons, remarkably short. In addition, these favourable results in support of gemcitabin-based chemoradiation have never since been confirmed by subsequent, more extensive randomised trials (Table 2), but chemoradiation with gemcitabine is widely used.

Although several trials since have tried to reproduce the results from 2003, with larger trials and more patients, confirmation of these results has so far been unsuccessful. In 2013, Mukherjee published a study randomising patients to chemoradiation concomitant with either capecitabine or gemcitabine [36]. Prior to randomisation, all initial 114 patients had received induction chemotherapy (gemcitabine and capecitabine). If progressing, they were not eligible for random allocation; therefore, only 74 patients received chemoradiation with either capecitabin or gemcitabine. Patients receiving capecitabine concomitant with radiation had a non-significantly longer median overall survival of 15.2 months compared to the gemcitabine arm (13.4 months) [48].

In Table 2, we present several trials that have tested chemoradiation to find the best regimen for this approach. Most trials in the table demonstrate a median overall survival of above 12 months. An average of 42 patients were included in each treatment arm in the eight randomised trials in Table 2, and the average overall survival was 11.9 months.

There is a mixture of patients with brPC and LAPC included in these trials, and therefore it is difficult to make any overall conclusion because the stage has a substantial effect on survival and resectability. 

Conclusions

- There is insufficient data to recommend the optimal concomitant drug with radiotherapy.
- The data are not sufficient to recommend which chemotherapy to use as induction therapy before chemoradiation.

**Table 2:** Induction therapy in patients with pancreatic cancer, not considered candidates for immediate resection—summary of randomised trials testing systemic therapy concomitant with radiotherapy (chemoradiation).

	Stage	Incl	Therapy	Radiotherapy Gy/fx	n	RR %	Res %	Median OS Months	2 Year OS %
Li [47]	LAPC	98-01	CRT (5FU) ⟹ Gem	50.4/28	16	13	-	6.7	0
IJROBP 2003			CRT (Gem) ⟹ Gem	50.4/28	18	50	-	14.4	15
Chung [49]	LAPC	97-02	CRT (Gem + DF) ⟹ GemDF	45/25	22	18	5	12	7 ^1^
IJROBP 2004			CRT (Pac + DF) ⟹ GemDF	45/25	24	25	8	14	15 ^1^
Wilkowski [50]	brPC	02-05	CRT (5FU)	50/25	30	19	13	9.6	5 ^1^
BJC 2009	LAPC		CRT (GemCis)	50/25	32	22	25	9.3	7 ^1^
			CRT (GemCis) ⟹ GemCis	50/25	31	13	19	7.3	16 ^1^
Landry [51]	brPC	03-05	CRT (Gem)	50.4/28	10	10	30	19.4	32 ^1^
JSO 2010			GCF ⟹ CRT (5FU)	50.4/28	11	18	18	13.4	15 ^1^
Mukherjee [48]	LAPC	09-11	GemCap ⟹ CRT(Gem)	50.4/28	57	23	5	13.4	9 ^1^
Lancet Oncol 2013			GemCap ⟹ CRT(Cap)	50.4/28	57	19	8	15.2	0 ^1^
Herman [52]	brPC	05-10	CRT (5FU) ⟹ Gem	50.4/28	90	12	11	10.0	10
JCO 2013	LAPC		CRT (5FU + TNF) ⟹ Gem	50.4/28	187	8	10	10.0	11
Su [53]	brPC	13-19	GOFL ⟹ CRT(Gem)	50.4/28	28	14	19	17.9	32
BJC 2022	LAPC		FOLFIRINOX ⟹ CRT(5FU)	50.4/28	27	22	4	19.2	30
Lierman [54]	LAPC	05-07	CRT (Gem + Cx) ⟹ Gem	54/25	35	NR	9	11.9	15
CTRO 2022			CRT (Gem + Cx) ⟹ GemCx	54/25	33	NR	33	14.2	27

^1^ Estimated 2 year overall survival.

### 3.3. Chemotherapy or Chemoradiation

Randomised trials comparing chemoradiotherapy (with or without induction chemotherapy) to systemic therapy alone have for decades demonstrated conflicting results. The current strategy is to start with combination chemotherapy, and there is an ongoing discussion as to whether patients with LAPC benefit from add-on chemoradiation. The rates of severe adverse events are consistently higher with chemoradiation compared to chemotherapy alone [55]. With the introduction of gemcitabine in the late 1990s, the strategy shifted to initiate chemotherapy alone since no study had shown the superiority of chemoradiation. However, retrospective studies suggested that chemotherapy administered before chemoradiation could improve the results further [27]. Such a treatment strategy could add to selecting those patients who could potentially benefit from chemoradiation and spare patients with rapidly progressive disease from toxicity. In 2007, Huguet et al. published a retrospective analysis of 181 patients with LAPC enrolled in the French prospective GERCOR studies and found that chemoradiation after initial chemotherapy prolonged the median overall survival to 15.0 months compared to chemotherapy alone (median overall survival 11.7 months). Despite the lack of data from randomised trials, initial chemotherapy followed by chemoradiation in non-progressive patients with good performance became an often-used strategy.

Regardless of the treatment strategy, the average overall survival for these patients remains low, especially for unselected patients. In a retrospective evaluation of 14,331 non-metastatic but unresected patients with PC registered in the US National Cancer Data Base (NCDB) between 2004 and 2012, the median overall survival was 9.9 months for patients receiving chemotherapy (monotherapy 8.8 months and combination chemotherapy 11.4 months) and 10.9 months for patients receiving chemotherapy combined with external beam radiotherapy [56]. However, it is important to note that this cohort was not a well-defined group of patients with LAPC.

The results of randomised trials of LAPC patients comparing chemoradiation with chemotherapy are inconsistent (Table 3), and two recent trials demonstrate opposite results [57,58]. In the French FFCD/SFRO 2000-01 trial, 119 patients (of 176 planned) were randomly assigned to induction chemoradiation (60 Gy/30 fractions with concomitant 5FU infusion and cisplatin) followed by maintenance gemcitabine or gemcitabine alone. The overall survival was shorter in the chemoradiation arm than in the gemcitabine arm (13.0 versus 8.6 months, HR 0.69) [57]. The ECOG trial randomised 71 patients (of 316 planned) to chemoradiation (50.4 Gy/28 fractions with concomitant gemcitabine) followed by maintenance gemcitabine or gemcitabine alone and found that chemoradiation significantly prolonged overall survival (11.1 versus 9.2 months, HR not reported). More chemoradiation patients had severe adverse events (41% vs. 9%), but there was no difference in the quality of life (QoL) [58].

The LAP-07 trial was planned after the retrospective French trial showed the benefit of adding chemoradiation to chemotherapy in selected patients [25,27]. The authors wanted to combine the advantages of both chemotherapy and chemoradiation by adding chemoradiation for LAPC patients showing no sign of progressive disease following four months of chemotherapy alone. The LAP-07 trial included 442 patients with LAPC. Patients were administered four months of gemcitabine (with or without erlotinib, first randomisation), and after four months of systemic therapy, 269 patients were randomised to either two supplementary months of gemcitabine or chemoradiation (3D-RT 54 Gy/30 fractions with concomitant capecitabine). An interim analysis was performed when 221 patients had died, reaching the early stopping boundaries for futility. The median overall survival from the date of the first randomisation was not significantly different between chemotherapy (16.5 months) and chemoradiation (15.2 months). The median overall survival for all 233 patients receiving gemcitabine was 13.6 months and 11.9 months for 219 patients receiving gemcitabine plus erlotinib. Chemoradiation was associated with a decreased risk of local progression (32% vs. 46%, *p* = 0.03), and there was no increase in severe toxicity except for nausea.

In Table 3, we summarise the trials that have tested a strategy with chemotherapy followed by chemoradiation. An average of 114 patients were included in each treatment arm in the randomised trials in Table 3; the average overall survival was 14.5 months.

In the largest trial so far, the German CONKO-007, 525 patients were enrolled between 2013 and 2021. Due to insufficient recruitment (the first calculations estimated the inclusion of 830 patients), the primary endpoint was changed from overall survival to R0 resection. After three months of induction chemotherapy (physicians choice: 15% gemcitabine, 85% FOLFIRINOX), 336 patients (64%) without progression were randomly assigned to continue chemotherapy for another three months or receive chemoradiation (with gemcitabine), and 60/167 patients (36%) and 62/169 (37%), respectively, had a resection. R0 resections were significantly higher in patients who received CRT (69% vs. 50%, respectively, *p* = 0.04), and the pathologic complete remission rate was higher in the CRT arm (18% vs. 2%, *p* = 0.004). Resected patients had a significantly longer median overall survival (19 vs. 14 months, *p* < 0.001). The median overall survival was 26 months in patients who had R0 resection. The effect of chemoradiation on resectability did unfortunately not translate into a prolonged overall survival. For all randomised patients, the median overall survival was 15 months in both arms (HR 0.98) [60].

Chemoradiation increases the R0 resection rate in surgically treated patients. We agree with the authors of CONKO-007 that a strategy of induction chemotherapy followed by chemoradiation and resection is achievable and that the combination selects a favourable subgroup, but we also have to learn to better select patients who benefit from this combined strategy. 

Conclusions

- There are conflicting results concerning the optimal treatment strategy
  (chemotherapy or chemoradiation) in patients with LAPC.
- One randomised trial was in favour of chemotherapy, and one trial was in favour of chemoradiation. Subsequent randomised trials did not show a benefit of supplementary chemoradiation; thus, no consensus on the optimal strategy in un-selected patients has been reached.
- Chemoradiation after induction chemotherapy increases the chance for R0 resection (and pathologic complete remission rate), but this benefit did not translate into a prolonged overall survival.
- Chemoradiation decreases the risk of local progression and is an alternative to the continuation of chemotherapy.

### 3.4. Systemic Therapy

For comparison, we also report data from randomised studies evaluating the efficacy of chemotherapy in patients with LAPC. As previously stated, patients with LAPC and metastatic cancer were often pooled, and only a few randomised trials have tested the efficacy of chemotherapy exclusively in LAPC. In a review and meta-analysis of 13 mainly retrospective trials that evaluated the efficacy of FOLFIRINOX with or without radiotherapy in 315 patients with LAPC [24], the median overall survival from the start of FOLFIRINOX was 24.2 months. The pooled proportion of resected patients was 25.9% (range 0–43%). In a more recent review [7] of the optimal management of LAPC (patients with brPC were included in some trials), the pooled resection after FOLFIRINOX chemotherapy was around 30% (561 patients) and approximately 20% after nab-paclitaxel/gemcitabine treatment (207 patients). In a meta-analysis of FOLFIRINOX-based preoperative therapy for LAPC, Chen et al. found a median overall survival ranging from 10.0 to 32.7 months (average 21.3 months) in 14 studies [62].

In the Italian GISCAD trial [63], 124 LAPC patients were treated with either gemcitabine or nab-paclitaxel/gemcitabine followed by chemoradiation. The median progression-free survival was prolonged from 4.0 to 7.0 months (HR 0.72, *p* = 0.045), and the median overall survival was prolonged from 10.6 months to 12.7 months (HR 0.72, *p* = 0.07). Chemoradiation was an option, but only administered in 40 patients (32%). The objective response rate (ORR) was 5% with gemcitabine and 27% with nab-paclitaxel/gemcitabine (*p* < 0.001).

The German NEOLAP trial included 168 LAPC patients who received initial therapy with nab-paclitaxel/gemcitabine [64]. After two months of treatment without progressive disease, 130 patients (77%) were randomised to continue with two cycles of nab-paclitaxel/gemcitabine or switch to four cycles of FOLFIRINOX. There was no significant difference in the response rate, the resection rate (primary endpoint), or overall survival (Table 4).

Comparable results from the French PRODIGE 29 trial were presented at ESMO 2022 [65]. In PRODIGE 29, 171 LAPC patients received gemcitabine or FOLFIRINOX. The primary endpoint (progression-free survival) was significantly prolonged in the FOLFIRINOX arm (9.7 months versus 7.5 months, *p* = 0.03), but (maybe surprisingly) the authors found no difference in median overall survival (15.6 vs. 15.1 months).

JCOG1407 randomised 126 LAPC patients to FOLFIRINOX or nab-paclitaxel/gemcitabine [51]. There was no significant difference in efficacy, but patients treated with nab-paclitaxel/gemcitabine obtained a slightly higher ORR (41% vs. 31%), and patients treated with FOLFIRINOX had a longer median overall survival (24 vs. 21 months) and a higher two-year survival rate (48% vs. 40%). 

Conclusion

- Patients with LAPC benefit more from combination therapy than gemcitabine monotherapy, but more prospective trials are required.

### 3.5. Chemoradiotherapy or Stereotactic Body Radiotherapy

Presently, radiotherapy is mainly used to maintain tumour control after chemotherapy and induce sufficient shrinkage (often after combination chemotherapy) to convert/down-size the primary non-resectable tumour to a resectable stage. Standard chemoradiation with a biological effective dose (see definition below) of 50–54 Gy after chemotherapy has had minimal impact on survival for patients with LAPC, as demonstrated previously in the current review [25,57,58]. Doses limited to approximately 50 Gy were originally established based on large fields and the tolerability of, e.g., the duodenum. Dose intensification of radiotherapy, including dose escalation or altered fractionation, has been studied in many malignancies. In a recent systematic review, it was concluded that radiotherapy intensification might improve the local control and overall survival in patients with head and neck and lung cancers, but the benefits were generally limited to studies testing high-dose radiotherapy without concurrent chemotherapy [67]. Higher doses did not improve outcomes in patients with oesophagal or rectal cancer. Pancreatic cancer patients were not included in the review because there are very limited data to support a dose–response effect with the use of conventional chemoradiation or radiotherapy. However, the observed long-term survival benefit observed in lung cancer, where SBRT may eradicate tumour cells and result in a long-term overall survival [67], has led to the use of SBRT in selected patients with LAPC.

Retrospective analyses in LAPC suggest that higher doses with a biologically effective dose (BED) of more than 70 Gy improve outcomes [68,69,70,71]. A BED is often used to assess effects in studies evaluating different fractionation regimens. The BED is a method to quantify treatment expectations when different fractionation regimens or cumulative doses are used. The BED is defined by the following equation: BED=nd(1+d/αβ), where n is the number of fractions, d is the radiation dose per fraction (in Gy), and αβ (actually the α/β ratio) describes the tissue radiation sensitivity where PDAC has been estimated to have an αβ of around 10 [34].

### 3.6. Stereotactic Body Radiotherapy

Dose escalation has become possible with the arrival of more advanced radiation delivery techniques such as SBRT. SBRT, also known as stereotactic ablative radiotherapy (SABR), is a highly focused radiation treatment that delivers ablative doses of radiation concentrated on a tumour. In SBRT, a high radiation dose is delivered in a few fractions (often five or less) to a limited target volume with high precision. The treatment is delivered either by using implanted markers when treating using a conventional accelerator or by using online MR guidance to localise the target at the treatment machine, permitting the use of small safety margins during treatment planning. The superior soft tissue contrast of MR images allows for daily treatment adaptation and thus allows very narrow margins, leading to a reduced radiation dose to adjacent healthy tissue. Therefore, SBRT offers a potential advantage in pancreatic cancer because of the possibility of delivering ablative doses without severe side effects and thereby possibly overcomes the radio resistance [68]. In addition, SBRT can be completed within 1–2 weeks, allowing the patient to continue effective systemic treatments without undue delay. Many mainly single-institution retrospective studies have shown the promising outcomes of SBRT, with local control rates from 50 to 100% and higher R0 resection rates for those who ultimately are able to undergo resection. To the best of our knowledge, the outcomes of SBRT in LAPC have never been compared with conventional chemoradiation in a randomised trial. Still, retrospective data have shown a longer median overall survival in patients receiving chemotherapy followed by SBRT compared to chemotherapy alone or chemotherapy followed by conventional chemoradiation [56,72] (Table 5).

SBRT is often preceded and/or followed by chemotherapy. Please note that patients starting chemotherapy before SBRT have an inherently longer median overall survival because patients with immediate or fast progression are not offered SBRT.

One of the first studies to test SBRT in LAPC was a small Danish phase II study in 2005. Twenty-two patients received SBRT in the form of 45 Gy in only three fractions (BED 112 Gy) over 5–10 days. The study demonstrated an overall survival of only 5.7 months with substantial toxicity, and the authors concluded that SBRT was not favourable for PC [73]. In 2008, Schellenberg et al. treated 16 patients with gemcitabine and SBRT prescribed as 25 Gy in a single fraction (BED 88 Gy). The median overall survival was 11.9 months. Acute toxicity was minimal, but late toxicity was substantial, e.g., duodenal ulcers [74]. Schellenberg et al. performed a similar study in 2011, and though they optimised the technique, the outcomes and adverse events were not improved [75].

In 2010, Mahadevan et al. presented a retrospective study testing SBRT (total dose 24–36 Gy in three fractions) in 36 patients with LAPC. Following SBRT, the patients received gemcitabine monotherapy for six months or until progression or intolerable side effects. The median overall survival was 14.3 months with limited toxicity [83]. Due to a high rate of distant metastasis in the previous study, Mahadevan and colleagues published a retrospective study in 2011, where 47 patients with LAPC received gemcitabine prior to SBRT. Patients without progression after two months of gemcitabine received SBRT (24–36 Gy/3 fractions). Eight of the initial forty-seven patients had metastatic progression and did not receive SBRT. The median overall survival for patients treated with SBRT was 20 months [84].

Herman et al. published a phase II study in 2015 evaluating gemcitabine and SBRT for patients with non-resectable LAPC. A total of 49 patients were included, receiving one cycle of gemcitabine over four weeks, followed by SBRT (33 Gy in five fractions). After SBRT, gemcitabine was continued until progression or untolerable toxicity. The median overall survival was 13.9 months. Rates of acute and late toxicities were the primary endpoints and showed limited acute toxicity, but 11% of patients had late toxicity of grade II or more [76].

In 2021, Teriaca et al. presented long-term outcome data from a phase II study of SBRT after FOLFIRINOX for LAPC, the LAPC-1 trial. Fifty patients were included in the study. All patients were to receive eight cycles of induction chemotherapy as FOLFIRINOX. If there was no sign of disease progression after eight cycles, patients were treated with SBRT (40 Gy in eight fractions). Eleven patients progressed during initial treatment and were not offered SBRT. The remaining 39 patients received SBRT. This study found a median overall survival of 18 months in the subgroup of patients treated with SBRT. Four patients experienced severe adverse events during SBRT [79].

The A021501 study randomised patients with brPC to eight cycles of preoperative FOLFIRINOX or seven cycles of FOLFIRINOX followed by hypofractionated image-guided radiation therapy (HIGRT) radiotherapy (n = 56), either SBRT (33–40 Gy in five fractions) or radiotherapy (25 Gy in five fractions). The study prematurely closed the experimental arm after an interim analysis showed that only 10 (at least 11 were expected) of the first 30 patients receiving FOLFIRINOX and radiotherapy underwent R0 resection. Severe adverse events were mainly observed during FOLFIRINOX, and only three patients (7%) experienced a grade 3 (no grade 4 or 5) adverse event that was at least possibly related to radiotherapy. The only pathologic complete responses occurred in the radiotherapy group (n = 2; 11%), and these facts make it even more challenging to understand the results. There have been concerns about the heterogeneity and delay of surgery in patients receiving radiotherapy [85,86].

One of the more recent studies published regarding ablative radiation therapy is a study by Tringale et al. Thirty patients were included in the study; the majority of patients being patients with LAPC (73%). All 30 patients had received chemotherapy prior to SBRT (50 Gy in five fractions). The median overall survival from diagnosis was not reached, with 1 and 2 year overall survival rates of 96.4% and 70.8%, respectively. No grade 3 or higher toxicities were described [87].

In conclusion, radiotherapy does benefit some individuals with LAPC and may even prolong the survival of selected patients. However, so far, there is very little high-level evidence to support the use of radiotherapy as a standard treatment in patients with LAPC. We need to learn how to select patients, and fortunately, there are ongoing randomised trials testing SBRT after up-to-date induction chemotherapy (Table 6). 

Conclusions

- SBRT is safe and well tolerated, especially with the use of daily dose adaption.
- SBRT is associated with improved local control.
- SBRT is less time consuming.
- There is a lack of randomised studies to support the use of SBRT in LAPC.

## 4. Discussion

In this review paper, we have presented and discussed the indications and effects of radiotherapy in patients with non-resectable pancreatic cancer. Radiotherapy in LAPC is controversial but nevertheless widely used. Except for SBRT (for which randomised trials are lacking), we focused on randomised trials, which so far have failed to demonstrate an unequivocal overall survival benefit when comparing chemoradiation with chemotherapy alone. Several of these randomised trials summarised in this review used outdated radiation techniques, which might account for at least some of this lack of efficacy.

As combination chemotherapy has improved the overall survival for patients with LAPC, local control of the primary cancer and preventing local relapses after resection might be of higher importance than before when the survival was short. It has been shown that local progression is associated with pain, nausea, weight loss, and a poor quality of life. Therefore, future trials investigating the role of radiotherapy should also include the quality of life of the patients.

Due to new modalities in radiotherapy, like online MR-guided radiotherapy, there is an increasing interest in SBRT. SBRT may offer an improved overall survival compared to standard chemoradiation, although to the best of our knowledge, there are no studies directly comparing standard chemoradiation to SBRT.

With SBRT, we have the possibility to improve the therapeutic efficacy of radiation therapy by allowing for ablative doses to the target while sparing organs of risk. This increase in the BED is fundamental in the approach to achieve longer loco-regional control.

Recent studies have demonstrated improved local control rates, with limited toxicity due to new radiation techniques enabling potential ablative doses to the tumour [88].

Further studies into the use of SBRT for patients with LAPC are needed to optimise the efficacy of radiotherapy as well as to determine when and how to apply it to improve outcomes for these patients.

## Figures and Tables

**Table 3:** Summary of randomised trials testing chemoradiation versus chemotherapy in patients with non-resectable pancreatic cancer (borderline or locally advanced pancreatic cancer).

	Stage	Incl	Therapy	Radiotherapy Gy/fx	n	Res %	Median OS Months	2 Year OS %
Chauffert [57]	LAPC	00-05	Gem		60	5%	13.0	21 ^1^
Ann Oncol 2008			CRT (CF) ⟹ Gem	60/30	59	3%	8.6	15 ^1^
Loehrer [58]	LAPC	03-05	Gem		37	0%	9.2	4 ^1^
JCO 2011			CRT (Gem) ⟹ Gem	50.4/28	34	0%	11.0	11 ^1^
Hammel [25]	LAPC	08-11	Gem 16w ⟹ Gem		221	6%	16.5	14 ^1^
JAMA 2016			Gem 16 ⟹ CRT (Cap)	54/30	221	3%	15.2	20 ^1^
Ioka [59]	LAPC	11-13	CRT (S1) ⟹ Gem	50.4/28	51	4%	19.0	32
JJCO 2021			Gem ⟹ CRT (S1) ⟹ Gem	50.4/28	49	6%	17.2	19
Fietkau, CONKO-007 [60]	LAPC	13-21	Gem or FFX		64	36%	15.0	23 ^1^
ASCO 2022			Gem or FFX ⟹ CRT (Gem)	50.4/28	64	37%	15.0	25 ^1^
Hewitt, PILLAR [61]	brPC	13-15	FFX or GnP ⟹ CRT (Cap)	50.4/28	158	26%	14.9	27 ^1^
AS 2022	LAPC		FFX or GnP + HAPa ⟹ CRT (Cap)	50.4/28	145	23%	14.3	26 ^1^

^1^ Estimated 2 year overall survival.

**Table 4:** Summary of randomised trials testing induction chemotherapy in patients with LAPC.

	Stage	Incl	Therapy	n	RR %	Res %	Median OS Months	2 Year OS % ^1^
Cascinu [63]	LAPC	16-19	Gem ⟹ CRT(Cap)	61	5	2	10.6	25 ^1^
EJC 2021			GnP ⟹ CRT(Cap)	63	27	6	12.7	25 ^1^
Kunzman, NEOLAP [64]	brPC	14-18	GnP ⟹ GnP	84	22	36	18.5	30 ^1^
Lancet GH 2021	LAPC		GnP ⟹ FOLFIRINOX	84	17	44	20.7	40 ^1^
Ducreux, [65]	LAPC	-	Gem	86	-	3	15.6	28 ^1^
ESMO 2022			FOLFIRINOX	85	-	4	15.1	28 ^1^
Ozaka, JCOG1407 [66]	brPC	16-19	FOLFIRINOX	62	31	8	23.0	46
EJC 2023	LAPC		GnP	63	42	8	21.3	41

^1^ Estimated 2-year overall survival.

**Table 5:** SBRT in patients with LAPC—summary of important trials.

Author, Year	Stage	Phase	Therapy	Gy/fx	BED10	n	mOS Months	2Y OS %	Toxicity > Grade 3
Hoyer, 2005 [73]	LAPC	II	SBRT ^2^	45/3	112	22	5.7	0	18%
Schellenberg, 2008 [74]	LAPC	II	Gem SBRT ^2^ ⟹ Gem	25/1	88	16	11.9	18	19%
Schellenberg, 2011 [75]	LAPC	II	Gem ⟹ SBRT ^2^ ⟹ Gem	25/1	88	20	11.8	20	5%
Herman, 2015 [76]	LAPC	II	Gem ⟹ SBRT ^2^ ⟹ Gem	33/5	55	49	13.9	18	28%
Comito, 2017 [77]	LAPC	II	CT ⟹ SBRT ^2^	45/6	79	45	19	36 ^1^	0%
Heerkens, 2018 [78]	LAPC	II	SBRT ^3^	24/3	43	20	8.5	-	0%
Teriaca, 2021 [79]	LAPC	II	FOLFIRINOX ⟹ SBRT ^2^	40/5	72	39	18	26 ^1^	10%
Ejlsmark, 2022 [80]	LAPC	II	FOLFIRINOX ⟹ SBRT ^3^	50/5	100	31	16.3	20 ^1^	3%
Michalet, 2022 [81]	(LAPC)	II	CT ⟹ SBRT ^3^	50/5	100	30	19.1	-	0%
Bordeau, 2022 [82]	LAPC	II	CT ⟹ SBRT ^3^	50/5	100	52	15.2	36	8%
	brPC								

^1^ Estimated 2 year survival, ^2^ IMRT radiation therapy, ^3^ online adaption guided.

**Table 6:** Summary of important ongoing randomised trials in patients with LAPC.

Trial Number, Name	Stage	Phase	Therapy	RT Gy/fx	n	Primary Endpoint	Expected Completion
NCT04089150	brPC	RII	GnP or mFFX		120	Local control	2025
MASTERPLAN	LAPC		GnP or mFFX ⟹ SBRT	40/5			
NCT04331041	brPC	RII	Chemo ⟹ SBRT	50/5	42	PFS	2025
	LAPC		Chemo ⟹ SBRT + defactenib	50/5			
NCT04986930	LAPC	RII	mFFX		92	PFS	2024
SABER			mFFX ⟹ SBRT	35/5			
NCT05083247	brPC	RII	GnP or mFFX		256	DFS	2030
STEREOPAC			GnP or mFFX ⟹ SBRT	35/5			
NCT05585554	LAPC	-	Chemo		267	OS	2028
LAP-ABLATE			Chemo ⟹ SBRT	50/5			
NCT04881487	Recur	RII	Chemo		174	OS	2028
ARCADE			Chemo ⟹ SBRT	40/5			

## Supplementary Materials

The following supporting information can be downloaded at: https://www.mdpi.com/article/10.3390/curroncol30070499/s1, Supplementary A.

## Abbreviations

The following abbreviations are used in this manuscript:

PC	Pancreatic cancer
LAPC	Locally advanced pancreatic cancer
PDAC	Pancreatic ductal adenocarcinoma
mPC	Metastatic pancreatic cancer
rPC	Resectable pancreatic cancer
brPC	Borderline resectable pancreatic cancer
RT	Radiotherapy
IMRT	Intensity-modulated radiation therapy
SBRT	Stereotactic body radiation therapy
sRT	Standard conventionally fractionated radiotherapy
CRT	Chemoradiation
Adr	Adriamycin
CCNU	Alkylating agent (Methyl-CCNU)
5FU	5-Fluorouracil
SMF	Streptozocin/Mitomycin/5FU
BSC	Best supportive care
FOLFIRINOX (FFX)	Folinic acid/5FU/Irinotecan/Oxaliplatin
mFFX	Modified FOLFIRINOX
Gem	Gemcitabine
Pac	Paclitaxel
DF	Doxifluridine
GemCis	Gemcitabine/Cisplatin
Cx	Cetuximab
GCF	Gemcitabine/Cisplatin/5FU
TNFerade	Tumour necrosis factor
GOFL	Gemcitabine/Oxaliplatin/5FU/Leucovorin
GemCap	Gemcitabin/Capecitabine
S1	Teysuno
HaPa	HyperAcute-Pancreas algenpantucel-L
GnP	Gemcitabine/Nab-paclitaxel
Cap	Capecitabine
Incl	Time of inclusion
n	Number of included patients
RR	Response rate
Res	Resection
NR	Not reached
BED	Biologically effective dose
CT	Chemotherapy
