# Supplementary material for: Radiotherapy for Locally Advanced Pancreatic Adenocarcinoma—A Critical Review of Randomised Trials
Source: Curr Oncol. 2023 Jul 18;30(7):6820–37. doi: 10.3390/curroncol30070499 (PMC10378124; doi:10.3390/curroncol30070499)
Supplement: Supplementary file 1 [file curroncol-30-00499-s001.zip › curroncol-2482175-Supplementary.pdf]

Supplementary A:

#	Searches	Results
1	exp pancreas adenocarcinoma/	41169
2	(Pancrea* adj3 (adenocarcinoma or adeno-carcinoma or ductal carcinoma or duct adenocarcinoma or duct carcinoma or ductal adenocarcinoma or ductal carcinoma or duct carcinogenesis or duct carcinoma or duct cell adenocarcinoma or duct cell carcinoma or ductal adenocarcinoma)).mp. [mp=title, abstract, heading word, drug trade name, original title, device manufacturer, drug manufacturer, device trade name, keyword heading word, floating subheading word, candidate term word]	52096
3	1 or 2	52096
4	(Pancrea* adj3 (adenocarcinoma or adeno-carcinoma or ductal carcinoma or duct adenocarcinoma or duct carcinoma or ductal adenocarcinoma or ductal carcinoma or duct carcinogenesis or duct carcinoma or duct cell adenocarcinoma or duct cell carcinoma or ductal adenocarcinoma)).ti,ab,kf.	41177
5	radiotherapy/	208083
6	stereotactic radiosurgery/ or stereotactic body radiation therapy/	33129
7	(radiotherap* or stereotactic radiosurg* or stereotactic body radiation therap*).mp. [mp=title, abstract, heading word, drug trade name, original title, device manufacturer, drug manufacturer, device trade name, keyword heading word, floating subheading word, candidate term word]	749221
8	5 or 6 or 7	749221
9	3 and 8	5501
10	(radiotherap* or stereotactic radiosurg* or stereotactic body radiation therap*).ti,ab,kf.	362197
11	5 or 6 or 10	457579
12	1 or 4	51859
13	11 and 12	2867
14	limit 13 to (books or chapter or conference abstract or conference paper or "conference review")	1343
15	13 not 14	1524
